# Mapping the NFT revolution: market trends, trade networks, and visual features

**DOI:** 10.1038/s41598-021-00053-8

**Published:** 2021-10-22

**Authors:** Matthieu Nadini, Laura Alessandretti, Flavio Di Giacinto, Mauro Martino, Luca Maria Aiello, Andrea Baronchelli

**Affiliations:** 1 Department of Mathematics, City University of London, London, EC1V 0HB UK; 2 The Alan Turing Institute, British Library, 96 Euston Road, London, NW12DB UK; 3 Technical University of Denmark, DK-2800 Kgs., Lyngby, Denmark; 4 Department of Neuroscience, Catholic University of the Sacred Heart, Rome, Italy; 5 IBM Research, Cambridge, MA USA; 6 IT University of Copenhagen, Copenhagen, Denmark; 7 UCL Centre for Blockchain Technologies, University College London, London, UK

**Keywords:** Statistical physics, thermodynamics and nonlinear dynamics, Information theory and computation

## Abstract

Non Fungible Tokens (NFTs) are digital assets that represent objects like art, collectible, and in-game items. They are traded online, often with cryptocurrency, and are generally encoded within smart contracts on a blockchain. Public attention towards NFTs has exploded in 2021, when their market has experienced record sales, but little is known about the overall structure and evolution of its market. Here, we analyse data concerning 6.1 million trades of 4.7 million NFTs between June 23, 2017 and April 27, 2021, obtained primarily from Ethereum and WAX blockchains. First, we characterize statistical properties of the market. Second, we build the network of interactions, show that traders typically specialize on NFTs associated with similar objects and form tight clusters with other traders that exchange the same kind of objects. Third, we cluster objects associated to NFTs according to their visual features and show that collections contain visually homogeneous objects. Finally, we investigate the predictability of NFT sales using simple machine learning algorithms and find that sale history and, secondarily, visual features are good predictors for price. We anticipate that these findings will stimulate further research on NFT production, adoption, and trading in different contexts.

## Introduction

“WTF are NFTs? Why crypto is dominating the art market” is the title of the February 21, 2021 episode of *The Art Newspaper* podcast^1^, signalling both the impact of Non Fungible Tokens (NFTs) on the art world and the novelty they represent for most of the general public. The revolution is not confined to the art market. While NFT adoption in gaming has already reached a certain maturity, for example concerning the trade of in-game objects, different other industries, especially those involved with the production of digital content such as music or video, are experimenting with the technology. Overall, in the first four months of 2021, the NFT volume has exceeded 2 billion USD, ten times more than the entire NFT trading volume in 2020^2^.

So, what’s an NFT? An NFT is a unit of data stored on a blockchain that certifies a digital asset to be unique and therefore not interchangeable, while offering a unique digital certificate of ownership for the NFT^3^. More broadly, an NFT allows to establish the “provenance” of the assigned digital object, offering indisputable answers to such questions as who owns, previously owned, and created the NFT, as well as which of the many copies is the original. Several types of digital objects can be associated to an NFT including photos, videos, and audio. NFTs are now being used to commodify digital objects in different contexts, such as art, gaming, and sports collectibles. Originally NFTs were part of the Ethereum blockchain but increasingly more blockchains have implemented their own versions of NFTs^4^.

The first popular example of NFTs is CryptoKitties, a collection of artistic images representing virtual cats that are used in a game on Ethereum that allows players to purchase, collect, breed, and sell them on Ethereum^5^. In December 2017, CryptoKitties congested the Ethereum network^6^. By many considered a chief example of the irrationality driving the cryptocurrency market in 2017^7^, CryptoKitties remained the only popular example of NFTs for almost 2 years. In July 2020, the NFT market started to grow^2^ and attracted a huge attention in March 2021, when the artist known as Beeple sold an NFT of his work for $69.3 million at Christie’s^8^. The purchase resulted in the third-highest auction price achieved for a living artist, after Jeff Koons and David Hockney^9^. Several other record sales followed^10,11^: three Cryptopunks—a collection of 10,000 unique automatically generated digital characters—were sold at $11.8, $7.6, and $7.6 million dollars, respectively; the first tweet was sold at $2.9 million dollars; and the Auction Winner Picks Name, an NFT with music video and dance track, sold at $1.33 million dollars. The profitability of NFTs has motivated celebrities to create their own NFTs, with collectibles of NBA and famous football players getting sold for hundreds of thousands dollars^12^.

Research on NFTs is still limited, and focuses mostly on technical aspects, such as copyright regulations^3^; components, protocols, standards, and desired properties^13^; new blockchain-based protocols to trace physical goods^14^; and the implications that NFTs have on the art world^15,16^, in particular as they allow to share secondary sale royalties with the artist. Empirical studies aiming at characterizing properties of the market have focused on a limited number of NFT collections, such as, CryptoKitties^17,18^, Cryptopunks, and Axie^19^, or on a single NFT market, such as, Decentraland^19,20^ or SuperRare^21,22^. These analyses revealed that the digital abundance of NFTs in digital games has led to a substantial decrease of their value^17^, and that, even if NFT prices are driven by the prices of cryptocurrencies^19^, the NFT market could be prone to speculation^18,20^. Further, it was shown that NFTs valued by experts are more successful^21^, and that, based on 16,000 NFTs sold on the SuperRare market, the structure of the the NFT co-ownership network is highly centralized, and small-world-like^22,23^.

In this paper, we provide a first comprehensive quantitative overview of the NFT market. To this end, we analyse a large dataset including 6.1 million trades of 4.7 million NFTs in 160 cryptocurrencies, primarly Ethereum and WAX, and covering the period between June 23, 2017 and April 27, 2021. We start by analysing the overall statistical properties of the NFT market and its evolution over time. Then, we study the network of interactions between NFT traders, and the network of NFT assets. NFTs are further clustered based on their visual features. Finally, we present the results of regression and classification models predicting the occurrence of NFT secondary sales and their price.

We break down our analysis by NFT categories, which are classified by manual inspection, with references to the classification proposed by NonFungible Corporation^24^, a specialized company that track NFTs sales, and OpenSea^25^, one of the largest NFT marketplace. However, the exact classification of different categories in which NFTs are used is outside of the scope of the present paper. For example, *Art* objects can be in some cases classified as *Collectibles*, while some *Game* objects may present sophisticated aesthetic and cultural properties that may qualify them as *Art*.

## Results

### The NFT market

**Figure 1:**
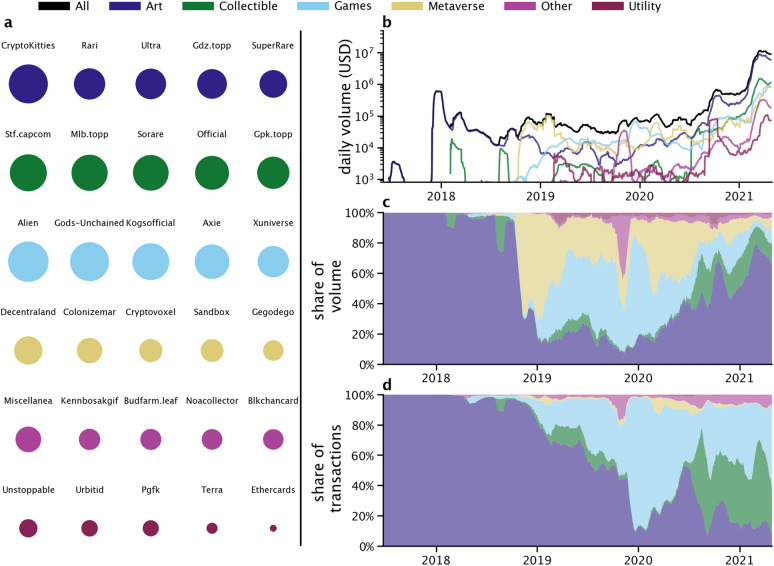
**Description of the NFT landscape.** (**a**) Top 5 NFTs collections (by number of assets) organized by category. The size of each circle is proportional to the number of assets in each collection. (**b**) Daily volume (in USD) exchanged over time for each category and for all assets (see legend). Days with volume below 1000 USD are not shown. (**c**) Share of volume traded by category. (**d**) Share of transactions by category. Results in these panels are averaged over a rolling window of 30 days.

Items exchanged on the NFT market are organized in *collections*, sets of NFTs that, in most cases, share some common features. Collections can be widely different in nature, from sets of collectible cards, to selections of art masterpieces, to virtual spaces in online games. Most collections can be categorised in six categories: *Art*, *Collectible*, *Games*, *Metaverse*, *Other*, and *Utility* (see also “SI”). We show the top 5 collections in terms of number of unique assets (*n*) for each category (see Fig. 1a).

Following an initial rapid growth in late 2017, when CryptoKitties collection gained worldwide popularity, the size of the NFT market has remained substantially stable until mid 2020, with an average of $\sim 60\,000$ US dollars traded daily (see Fig. 1b). Starting from July 2020, the market has experienced a dramatic growth, with the total volume exchanged daily surpassing $\sim 10$ million US dollars in March 2021, thus becoming 150 times larger than it was 8 months earlier.

We measured to what extent different NFTs categories contribute to the size of the whole NFT market. Until the end of 2018, the market was fully dominated by the *Art* category, and in particular by the CryptoKitties collection. From January 2019, other categories started gaining popularity, both in terms of total volume exchanged (see Fig. 1b,c) and number of transactions (see Fig. 1d). Overall, in the period between January 2019 and July 2020, $\sim 90\%$ of the total volume exchanged on NFT was shared by the *Art*, *Games*, and *Metaverse* categories, contributing $18\%$, $33\%$, and $39\%$ respectively. Starting from mid July 2020, the market volume has been largely dominated by NFTs categorized as *Art*, which, since then, have contributed $\sim 71\%$ of the total transaction volume, followed by *Collectible* assets accounting for $12\%$. Importantly, however, the market composition is quite different when considering the number of transactions. Since July 2020, the most exchanged NFTs belong to the categories *Games* and *Collectible*, which account for $44\%$ and $38\%$ of transactions. Instead, only $10\%$ of transactions are related to NFTs categorized as *Art*. Overall, we observe that the share of volume spent in *Art* has been growing since 2020, while its share of transactions has been decreasing (Fig. 1d). The discrepancy between volume and transactions reveals that prices of items categorized as *Art* are higher, on average, compared to other categories.

**Figure 2:**
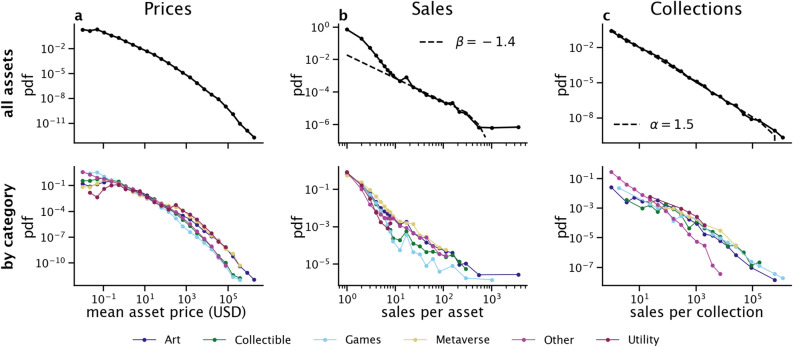
**Statistical properties of the NFT market.** (**a**) Distribution of the average price (USD) for all NFTs (top) and by NFT category (bottom). (**b**) Distribution of number of sales per NFT for all NFTs (top) and by category (bottom). The dashed line is a power law fit $P(s) \sim s^{\beta }$, with $\beta =-1.4$, where *s* is the number of sales. (**c**) Distribution of number of assets per collection for all NFTs (top) and by category (bottom). The dashed line is a power law fit $P(n) \sim n^{\alpha }$, with $\alpha =-1.5$, where *n* is the number of unique assets.

We dig further into these differences by looking at the distribution of NFT prices across categories (see Fig. 2a), which we find to be broadly distributed. We observe that the average sale price of NFTs is lower than 15 dollars for $75\%$ of the assets, and larger than 1594 dollars, for $1\%$ of the assets. Considering individual categories, NFTs categorized as *Art*, *Metaverse*, and *Utility* reached higher prices compared to other categories, with the top $1\%$ of assets having average sale price higher than 6290, 9485, and 12,756 dollars respectively. Note that these categories are different in sizes, so $1\%$ of assets corresponds to 8593, 472, and 78 NFTs in the *Art*, *Metaverse*, and *Utility* categories, respectively. The highest prices so far were reached by assets categorized as *Art*, with 4 NFTs that were sold for more than 1 million dollars.

To assess the market activity, we measured how often individual assets are traded. Here, we refer to the first time an asset is sold as the asset’s *primary sale*, and to all other sales as *secondary sales*. All assets considered in this study had a primary sale, but only $\sim 20\%$ of them had a secondary sale (see “SI”). We observe that the tail of the distribution of number of sales *s* per asset, for $s \ge 10$, is well characterized by a power-law function $P(s) \sim s^{\beta }$, with $\beta =-1.4$, estimated following^26^ (see Fig. 2b). When looking at different categories, the distribution of number of sales is affected by cut-off values. For example, the maximum number of sales for assets in the *Utility* category is 12, while an asset in the *Games* category is sold more than a thousand times, and an asset in the *Art* category more than five thousands times. Note that only $0.07\%$ of all assets are sold more than 10 times. Also, the size of collections *n* is well described by a power-law function $P(n) \sim n^{\alpha }$, with $\alpha =-1.5$ (see Fig. 2c), implying the distribution of sizes is broad. We find that $\sim 75\%$ of collections comprise less than 37 unique assets, and $\sim 1\%$ have more than 10,400 unique assets.

**Figure 3:**
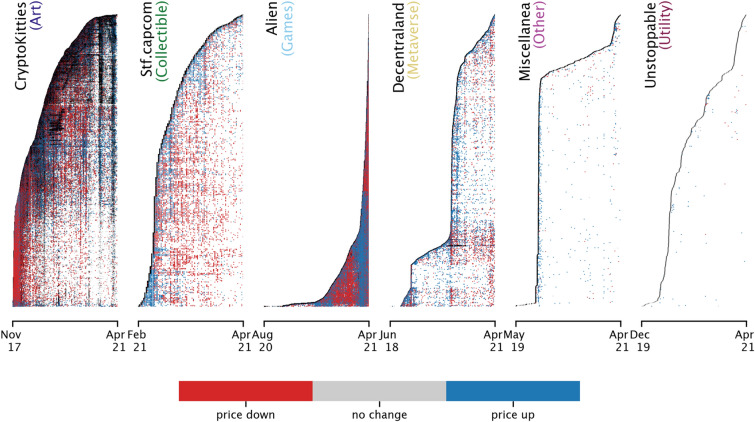
**Secondary sale prices.** Sales over time for the top collection in terms of number of sales in each NFT category (CryptoKitties, Stf.capcorn, Alien, Decentraland, Miscellanea, and Unstoppable). Each horizontal line represents an NFT and each dot a sale. Sales are coloured based on the change in price compared to previous sale (see colourbar).

Temporal patterns of secondary sales are unique for each collection, as evidenced by considering the top collection in each category (see Fig. 3). For example, when Cryptokitties emerged in 2017, secondary sale prices were typically lower than the price of their first sale. More recently in 2021, instead, their secondary sale prices have gone up because of an increase in the number of potential customers. Other collections, like Alien, alternated periods when secondary sale prices went down and period when they went up. In the Unstoppable collection secondary sales are rare because NFTs correspond to web domains secured by blockchain technology. In 2017, secondary sale price were lower than primary in 66% of the cases, while in 2021 only 27% of secondary sales had lower prices than the primary one.

### The networks of NFT trades

How do traders interact with each other? Are there central actors? We approach these questions adopting a network science approach^23,27^. We consider the *network of trades*, where nodes are traders, a directed link from a trader to another exists if the former (the buyer) purchases at least one NFT from the latter (the seller). Each link has a weight corresponding to the total number of items that the buyer bought from the seller.

**Figure 4:**
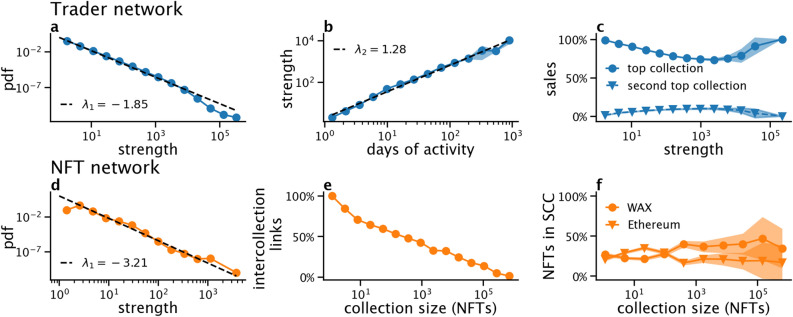
**Key network properties.** (**a**) Pdf of the traders’ strength. (**b**) Traders’ strength as a function of the number of days of activity. (**c**) Percentage of transaction traders make toward their top and second-top NFT collections. (**d**) Pdf of the NFTs’ strength. (**e**) Percentage of transactions between NFTs in different collections as a function of the size of the collection. (**f**) Percentage of NFTs belonging to the first and second largest strong connected component (SCC). Solid curves in (**b**), (**c**), (**e**) and (**f**) represent average values, while respective bands the 95% confidence interval.

First, we study the behaviour of individual NFT traders by focusing on properties of the nodes. We find that traders activity is highly heterogeneous: the strength of traders (nodes) *s*, defined as the total number of purchases and sales made by each trader, is distributed as a power law $P(s)\sim s^{\lambda _1}$ with exponent $\lambda _1 = -1.85$ (see Fig. 4a), such that the top 10% of traders alone perform 85% of all transactions and trade at least once 97% of all assets. Further, we find a superlinear relation between the strength of a trader and the total number of days of activity *d*, with $s\sim d^{\lambda _2}$ and $\lambda _2 = 1.28$ (see Fig. 4b). This result reveals that the average number of daily trades is larger for traders active over long periods of time. Traders are also specialized: measuring how individuals distribute their trades across collections, we find that traders perform at least 73% of their transactions in their top collection, while at least 82% in their top two collections combined. The relation between strength and specialization is not monotonic: the most specialized traders have either few (less than ten) or many (more than ten thousands) transactions (see Fig. 4c). A specialized trader is the one with Ethereum address “0xfc624f8f58db41bdb95aedee1de3c1cf047105f1”, that exchanges tens of thousands of CryptoKitties. Similar relationships hold when buying and selling behaviours are considered separately (see “SI”).

Secondly, we turn to properties of the network links, describing interactions between pairs of traders. We find that the distribution of link weights is well characterized by a power law distribution, with the top 10% of buyer–seller pairs contributing to the total number of transactions as much as the remaining 90% (see “SI”). An interesting question is whether traders connect preferentially to traders that have similar strength. We tackle this question by studying the assortativity coefficient *r*^28^, that measures the correlation between the sum of the weights of all outgoing links (the outgoing strength) of a given node with the average sum of the weights of incoming links (the incoming strength) of its neighbours. We find that the assortativity, which takes value $r=-0.024$, is close to the null value zero, implying that traders do not connect to other traders based on the similarity of their connection patterns.

Finally, we focus on the network structure. Building upon the result that traders are specialized, we assign each trader to their top collection, and we study the modularity^29^ of the network under this partition of nodes. The modularity is a metric bounded between $-0.5$ and 1, which is positive when the density of links among nodes assigned to the same partition is larger than it would be expected by chance. We find that the modularity *Q* of the collections partition is $Q = 0.613$, significantly higher than what expected from a random network $Q = 0.0823 \pm 0.0001$ (see “SI”). It reveals that the collections well represent the underlining network structure, where traders specialized in a collection tends to buy and sell NFTs with other traders specialized in the same collection.

We now turn to the exploration of how NFTs are connected to one another. To this end, we construct the *network of NFTs*, where nodes are NFTs and a directed link exists between two NFTs that are purchased “in sequence”, e.g. a link is created from an NFT to another when a buyer purchases the former and then the latter, with no purchases between the two (see “SI” for more details). Rather than linking all NFTs ever traded by the same trader, this choice allows to understand the relations between NFT that are semantically similar, because they are bought by the same trader in approximately the same period of time. Further, it ensures that the network structure is not dominated by large cliques.

The distribution of NFTs strength decays as a power law with exponent $\lambda _3 = -3.21$ (see Fig. 4d). Note that the strength of NFTs is different to the total number of sales per NFT (previously shown in Fig. 2b), due to how the network is constructed. In fact, when two NFTs are purchased simultaneously, this creates two links for each of the two nodes (one ingoing and one outgoing). The next question we ask is: which NFTs are connected to one another? We find that NFTs in small collections tend to be bought in sequence with NFTs in other collections (see Fig. 4e). On the contrary, NFTs in large collections, like CryptoKitties or Gods-Unchained, tend to be bought in sequence with NFTs in the same collection.

What are the implications of this behaviour on the NFT network structure? We investigate the relation between the structure of the NFT network and NFTs collections, by studying the modularity^29^ of the network under the partition of NFTs (nodes) into NFT collections. We find that the modularity *Q* of the collections partition is $Q = 0.80$, significantly higher than what expected from a random network $Q = 0.1110 \pm 0.0001$. It reveals that (1) the network is clustered and (2) the collections well represent the underlining community structure. By further exploring the relationship between traders’ behaviour and NFT networks structure, we unveil that, while the NFT network is clustered, communities are not isolated. That is, some traders buy or sell assets belonging to multiple collections. The network of NFTs has two strongly connected components (SCC)^30^, defined as groups of nodes such that, starting from a given NFTs, it is possible to reach any other NFTs in the SCC following directed links. The largest SCC include NFTs traded in the WAX blockchain, consisting of 35% of all NFTs, while the second largest includes NFTs traded in the Ethereum blockchain, consisting of 20% of all NFTs (see Fig. 4f). While the high network modularity reveals that traders tend to purchase assets from the same collection in sequence, the presence of very large SCCs reveals that there are less frequent sequences of purchases in different collections.

**Figure 5:**
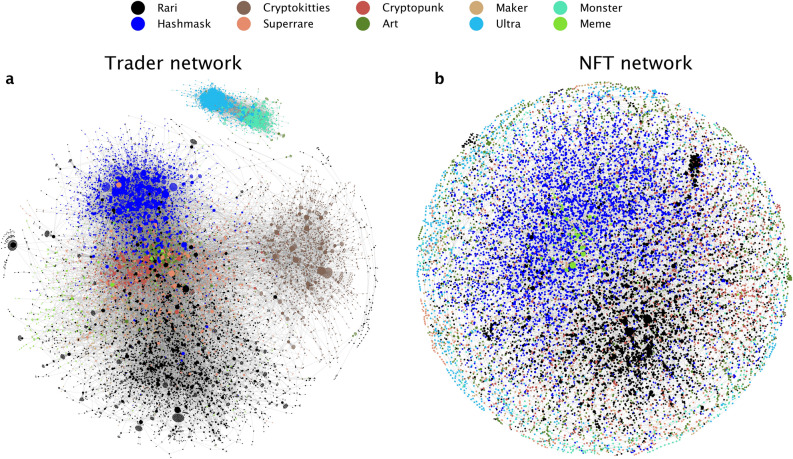
**Networks visualization.** (**a**) Trader network, where nodes represent traders and links sales between a pair of them. (**b**) NFT network, where nodes represent NFTs and links when a pair of NFTs is purchased in “sequence”. For visualization purposes, we selected the ten top collections in the *Art* category on February 2021. Visualization is done using Netwulf^31^.

A visual representation of the trader network including the *Art* category on February 2021 shows the clusters formed by NFT traders specialized in the same collection (see Fig. 5a). Similarly, the same visualization for the NFT network shows a similar trend, where NFTs, albeit surrounded by other NFTs in the same collection, tend to form a sparser structure (see Fig. 5b).

We then study the networks consisting of assets in the same category and blockchain (see “SI”). We find that key results presented above, including the shape of the strength distributions, hold across categories. Also in this case, we find that traders, independently from the category considered, are specialized: the fraction of individual trades in the top collection is included between 59%, for the *Other* category, and 98%, for the *Utility* category. Similarly, the fraction of individual trades in the top collection is 70% for the WAX blockchain and 91% for the Ethereum blockchain category. Relative to the number of total NFTs in each category, the WAX component contains 55.0% of all NFTs labeled as *Collectible*, but only the 0.06% of all NFTs labeled as *Utility*. On the contrary, the Ethereum component has the 54.8% of all *Art*, but only the 10.6% of *Games*.

### Visual features

NFTs are linked to digital assets of different types, including videos, text, animated GIFs, and audio. Currently, the most popular NFTs are images^10,11^. We select NFTs associated with images and take a snapshot of animated GIFs, and analyse them with the pre-trained convolution neural network AlexNet. AlexNet extracts from an image a vector of 4096 values that is a dense representation of the image’s visual features. With this representation, vectors extracted from images that are visually similar are close in the vector space. To quantify the visual difference between pairs of pictures, we calculated the cosine distance (CD) between them, a value that goes from zero (for identical images) to one (for highly different images). We measured such distance between pictures within the same collection and across collections.

**Figure 6:**
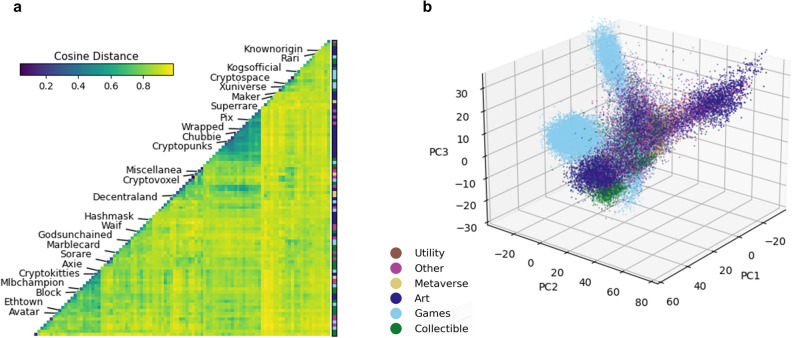
**Visual features representation.** (**a**) Cosine distance of graphical digital objects between items grouped by collections and categories (coloured bands on the right), recognising aesthetical similarities and uniformity between and within these groups. For visualization purposes, we selected the largest 98 collections in our dataset. (**b**) The dimensionality reduction of AlexNet vectors by PCA and their visualization in the PC1, PC2 and PC3 space, broken down by NFT categories, demonstrate the presence of graphically uniform clusters. For visualization purposes, we downsampled the digital objects associated with the CryptoKitties and Sorare collections, which alone constitute the 61% of the whole dataset.

The average CD calculated between items which belong to the same collection is significantly lower ($\mu = 0.59$, $\sigma = 0.20$) compared to the one obtained for objects from two different collections ($\mu = 0.87$, $\sigma = 0.06$), confirming an intra-collection graphical homogeneity. Figure 6a shows the matrix of average CD values between all pairs of collections. Values on the diagonal represent the intra-collection CD values, and reveal that most collections have a high degree of homogeneity (e.g., Sorare (CD = 0.24) or Cryptopunks (CD = 0.33)) but some are more heterogeneous (Rarible (CD = 0.89)). In short, many collections have their own style, graphical hallmarks that distinguish them from others. There are also sub-groups of collections, usually within the same category (coloured band in Fig. 6a), which share some common visual features. This is the case for collections containing pieces of pixel-art, including Chubbie, Cryptopunks and Wrapped Punks, or the similarities observed between Cryptokitties and Axie.

To map the images into a lower-dimensional feature space that can be used in practice for prediction and visualization, we apply Principal Component Analysis (PCA) to the AlexNet vectors. PCA uses linear combinations of the 4096-dimensional vectors to project them into vectors with an arbitrarily lower number of dimensions and such that the variance of datapoints in the projected space is maximized. Considering the whole sample, which consists of about 1.25 million graphical objects, the first five principal components explain together about the 38.3% of the total variance, progressively distributed from PC1 to PC5 as follow: 20.3%, 7.3%, 4.0%, 3.8% and 2.7%. The PC1 to PC5 scores are used to test the capacity of visual features for predicting sales (see next subsection), while PC1, PC2, and PC3 for visually representing the data through a 3D scatter plot and showing intra-categories homogeneity (see Fig. 6b). This can be quantified by looking at the average Euclidean distance in the PC1, PC2, PC3 space between objects of the same category and comparing it to the one calculated among objects of different categories. Considering the whole sample and calculating the distance between all the points, the average value obtained between elements of different categories is 1.67 bigger than for elements of the same category. However, as we already described for the cosine distance in the AlexNet vector space, this is mainly due to the intra-collections homogeneity, as demonstrated calculating the average inter-collection distance which results more than three times (3.17) bigger than the intra-collection distance and secondarily to the presence of intra-categories clusters of similar looking collections. This is most likely caused by the market responsiveness to the success of a collection, which induces other creators to follow the trend and offer variations on the theme.

### Predicting sales

To identify the factors associated with an NFT’s market value, we fit a linear regression model to estimate the price of primary and secondary sales from different sets of features, calculated considering only the data preceding the day of the NFT’s primary sale. The features (whose detailed formulations are provided in “SI”) include the degree and PageRank centrality of the buyer and seller in the networks of NFT trades ($k_{buyer|seller}$, $PR_{buyer|seller}$), the principal components of visual features of the object linked to the NFT ($vis_{PCA_{1 \ldots 5}}$), a prior probability of sale within the collection ($p_{resale}$), and the past median price of primary and secondary sales within the collection ($median\,price$).

NFT’s price correlates strongly with the price of NFTs previously sold within the same collection (see “SI”). The median sale price of NFTs in the collection predicts more than half of the variance of price of future primary and secondary sales. The prediction is more accurate when the median of the past sale price is calculated over a recent time window preceding the primary sale, e.g., the prior time window of one week is better than considering the entire time window preceding the NFT’s primary sale. Similar results, albeit with generally lower correlations, are found when the secondary sale price is the object of the regression (see “SI”). As one would expect, the price of secondary sales is strongly correlated with the price of primary sale, and the predictive power of the variables declines as one attempts to cast a prediction over longer periods of time: $R_{adj}^2=0.90$ when predicting the median secondary sale price over the next week, and falls to $R_{adj}^2=0.77$ when extending the prediction over the next 2 years (see “SI”). A similar relation is found between the secondary sale price and the median price of the NFTs collection (see “SI”).

**Figure 7:**
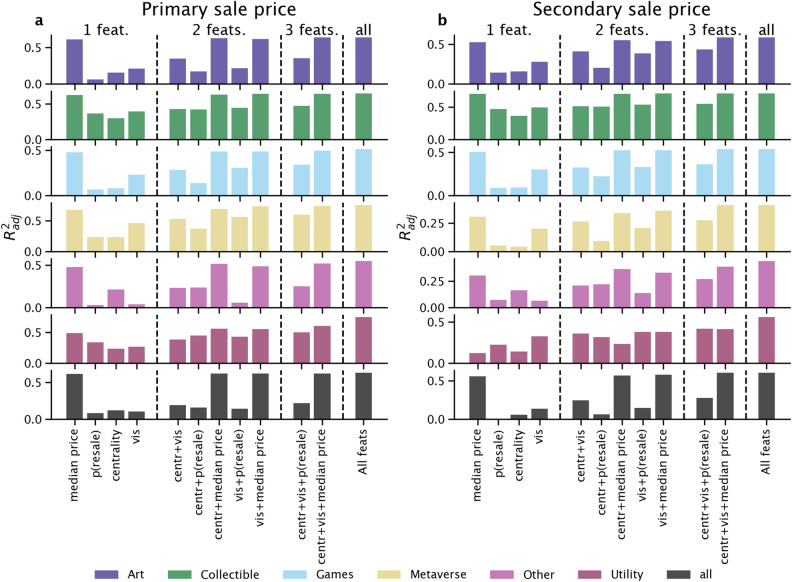
**Regression results.** $R_{adj}^2$ of a linear regression fit to predict the primary sale price (**a**) and the secondary price sale 1 month after the primary sale (**b**) from different sets of features. Results are broken down by NFT categories. The abbreviation “feat(s).” stands for “feature(s)”.

Other features than prior sale history are predictive of future primary sale price (see Fig. 7a) and median secondary sale price (see Fig. 7b). Centrality measures of the buyer and seller in the trader network ($R_{adj}^2\in [0.05,0.12]$) and visual features of the object linked to the NFT ($R_{adj}^2 \in [0,0.08]$) explain roughly one-fifth to one-fourth of the variance when used in combination ($R_{adj}^2 \in [0.18,0.25]$). When considered in combination with the median price of previous sales, they increase the predictive power by almost 10% for the secondary sale price ($R_{adj}^2$ from 0.55 to 0.6). When fitting separate regressions for each category, it becomes apparent that the predictability of future prices and the predictive power of different sets of features varies depending on the NFT category. The *collectible* category is the easiest to predict, with centrality and visual features yielding $R_{adj}^2 \in [0.30,0.36]$ and $R_{adj}^2 \in [0.40,0.50]$, respectively. These two families of features have the largest compound effect in the *Art* category; in the secondary sale price prediction, centrality features boost the predictive power of visual features by more than 50%. Regression coefficients of individual features for the task of secondary sale price prediction one month after the primary sale are presented in Table 1.

**Table 1:** Secondary sale price prediction. Linear regressions to predict the NFTs’ median secondary sale price one month after their primary sale from three families of features: centrality on the trader network (*k*, *PR*), history of sales in the NFT’s collection (namely prior probability of secondary sale $p_{resale}$ and median sale price 1 week before the sale *medianprice*), and visual features ($vis_{PCA_i}$). Regression models were fit to different categories of NFTs independently. For each category, the number of NFTs and collections it contains is reported. The $R_{adj}^2$ is a measure of goodness of fit, and it quantifies the proportion of the data variance explained by the model. The p-values of all $\beta$ coefficients are $<0.01$ except for those marked with $^{\bullet }$, which are all $>0.05$.

Feature	$\beta$ coefficients						
	All	Art	Collectible	Games	Metaverse	Utility	Other
const.	$-0.029$	0.030	$-0.086$	$-0.181$	0.210	2.054	0.149
$k_{buyer}$	$-0.018$	0.022	$-0.032$	$-0.132$	$-0.078$	$-0.010^{\bullet }$	$-0.207$
$k_{seller}$	$-0.166$	$-0.211$	0.000	0.026	0.166	$0.198^{\bullet }$	$-0.347$
$PR_{buyer}$	0.129	0.077	0.162	0.317	0.206	$-0.241^{\bullet }$	0.336
$PR_{seller}$	0.302	0.367	$-0.031$	$-0.066$	$0.009^{\bullet }$	$-0.382$	0.459
$p_{resale}$	0.029	$-0.041$	0.079	0.023	$0.046^{\bullet }$	0.465	$0.251^{\bullet }$
*Medianprice*	0.769	0.711	0.970	0.815	0.436	0.478	0.687
$vis_{PCA_1}$	0.098	0.153	0.049	0.174	0.175	$-1.136$	0.021
$vis_{PCA_2}$	$-0.120$	$-0.130$	$-0.044$	$-0.064$	$-0.669$	$-0.817$	$-0.181$
$vis_{PCA_3}$	0.019	0.027	0.063	0.203	$0.112^{\bullet }$	$-1.292$	$-0.037^{\bullet }$
$vis_{PCA_4}$	0.040	0.028	$-0.003^{\bullet }$	0.130	$-0.018^{\bullet }$	$-0.911$	$-0.116$
$vis_{PCA_5}$	0.063	0.018	0.276	0.102	0.296	$0.071^{\bullet }$	0.301
#NFTs	407,549	251,369	69,015	78,848	2693	314	5297
#Collections	3307	114	73	48	12	6	3054
$R_{adj}^2$	**0.6**	**0.589**	**0.709**	**0.535**	**0.408**	**0.562**	**0.44**

When predicting secondary sale prices, we consider only those NFTs that were sold in a secondary sale. These NFTs are the minority: less than 10% are sold at least once within one week after the primary sale, and only about 22% within 1 year (see “SI”). Using the same set of features that we selected for the price regression, we trained AdaBoost^32^, a binary classifier, to assess to what extent it is possible to predict whether an NFT will be sold after its primary sale (for more details see in “SI”). We find that this is possible to a certain extent. The prediction is most accurate when training and testing the classifier on *Art* NFTs only ($F1>0.8$), whereas the prediction is less reliable for the other categories ($F1 \in [0.14,0.33]$, see “SI”). The median price of the collection is among the strongest predictors, but not always the strongest. The prior probability of sale in the collection is also a strong signal, and centrality and visual features combined can sometimes outperform other feature combinations (e.g., in the *Metaverse* category). Last, the prediction is most accurate when trying to predict the occurrence of a secondary sale over longer periods of time (see “SI”).

## Conclusion

The NFT market is less than four years old and has boomed in 2021. This paper presented the first overview of some key aspects of it by looking at the market history of 6.1 million NFT trades across six main NFT categories including art, games and collectibles. In brief, (1) we analyzed the main properties of the market, (2) we built and studied the traders and NFTs networks and found that most traders are specialised, (3) we showed that NFT collections tend to be visually homogeneous, and (4) we explored the predictability of NFT prices revealing that, while past history is as expected the best predictor, also NFT specific properties, such as the visual features of the associated digital object, help increase predictability.

It is important to highlight the main limitations of our study, which represent also directions for future work. First, we gathered data from a variety of online NFT marketplaces and not directly from the Ethereum or WAX blockchains, so that we have likely missed a number of “independent” NFT producers. Second, we mostly adopted an accepted categorisation for the NFTs, which includes a number of arbitrary decisions and could however be further refined (as every categorization). Third, since our primary goal was to provide a general overview of the market, we did not extensively explore all the available methods e.g., for the features extraction from images^33^ and their clustering in a lower-dimensional space^34^, machine learning for price prediction^35^, or market modelling^36^. We also did not consider collective attention as measured e.g. from social media or Wikipedia, which can be a further source of information about market behaviour^37–39^. Fourth, we considered mostly the Ethereum and WAX blockchains, but several other platforms offer smart contracts and NFTs. Finally, our price prediction exercise did not include information about the creator of the (digital) object associated to the NFTs. While this is due mainly to the dataset, and in many cases the identity of the creator is not available or does not exist (e.g., for AI generated images), it is likely that in certain contexts, and specifically for art, this can be an important aspect to consider.

Overall, NFTs are a new tool that satisfies some of the needs of creators, users, and collectors of a large class of digital and non-digital objects. As such, they are probably here to stay or, at least, they represent a first step towards new tools to deal with property and provenance of such assets. We anticipate that our study will help accelerate new research on NFT in a broad array of disciplines, including economics, law, cultural evolution, art history, computational social science, and computer science. The results will also help practitioners make sense of a rapidly evolving landscape and inform the design of more efficient marketplaces as well as the associated regulation.

## Data and methods

We summarize our data collection below and provide a detailed description of our data manipulations in “SI”.

### Sales data collection

Our dataset includes only transactions representing purchases of NFTs, whose ownership change following that transaction. We exclude from our analysis any transactions representing the minting of NFTs or bids during an auction. We track different cryptocurrencies. Etherum blockchain data for the collections SuperRare, Makersplace, Knownorigin, Cryptopunks, and Asyncart were shared by NonFungible Corporation^24^, a company that tracks historical NFT sales data to build NFT valuations. Other Ethereum blockchain data were downloaded from four open-source APIs: CryptoKitties sales^40^, Gods-Unchained^41^, Decentraland^42^, and OpenSea^43^. With OpenSea that allows trading in multiple cryptocurrencies. We also monitored the WAX blockchain, through tracking transactions in the Atomic API^44^.

We group NFTs into six categories: *Art* consisting of digital artworks such as images, videos, or GIFs; *Collectible* representing items of interest to collectors; *Games* including digital object used in competitive games; *Metaverse* consisting of pieces of virtual worlds; *Utility* representing items having a specific function; and *Other* including the remaining collections. More details on the NFT categorization are explained in “SI”. The final, cleaned dataset includes 935 million USD traded in 6.1 million transactions involving 4.7 million NFTs grouped in 4624 collections. Our dataset includes transactions in 160 different cryptocurrencies with most of them made in WAX (52% of the total number of transactions), while the volume in USD is mostly ETH (81% of the total volume). We show general statistics of the categories of NFTs considered, involving a total of 359,561 buyers, 314,439 sellers, trading 4.7 millions NFTs involving 953 million USD in cryptocurrencies (see “SI”).

### Image collection and visual feature extraction

For each NFT in our dataset (except for less than 3000 exceptions) we managed to collect at least one URL that points to a copy of the NFT’s digital object. We focused only on objects with image file formats (e.g. PNG, SVG, JPEG) and GIFs, for a total of about 1.2 million unique graphical objects associated with 4.7 million unique NFTs. Note that a single digital object can be related to multiple NFTs; this happens for example for identical playing cards that are minted in multiple copies, each associated with a different NFT. Since our algorithm for visual feature extraction works with static images, we converted the animated GIFs to PNGs by extracting central frame of each GIF. In order to succinctly represent the visual features that characterize an image, we encode it into a latent space using a neural network. Specifically, we pick the PyTorch^45^ implementation of AlexNet^46^, a deep convolutional neural network architecture designed for image classification. We initialize AlexNet with weights pre-trained on ImageNet^47^, a widely-used reference dataset of labeled images. Given an image in input, AlexNet passes it through multiple layers of transformation. The second to last layer (i.e., the layer before the classification layer) is a vector consisting of 4096 values that constitute a dense representation of the input image into a high-dimensional space. These vectors can be used for a variety of tasks such as similarity ranking, clustering, or classification. To reduce the dimensionality of AlexNet vectors, we extracted their principal components using Principal Component Analysis (PCA)^48^, and selected the five most relevant ones. PCA projects each point of the high-dimensional space into a space with a desired number of dimensions, while preserving the data variation as much as possible.

## Supplementary Information

Supplementary Information.

## Data Availability

Data and scripts used to download from the different APIs are available at https://osf.io/wsnzr/?view_only=319a53cf1bf542bbbe538aba37916537.
