# Supplementary material for: Mapping the NFT revolution: market trends, trade networks, and visual features
Source: Sci Rep. 2021 Oct 22;11:20902. doi: 10.1038/s41598-021-00053-8 (PMC8536724; doi:10.1038/s41598-021-00053-8)
Supplement: Supplementary file 1 — Supplementary Information. [file 41598_2021_53_MOESM1_ESM.pdf]

Mapping the NFT revolution: market trends, trade networks, and visual features

Matthieu Nadini[1,2], Laura Alessandretti[3], Flavio Di Giacinto[1,4], Mauro Martino[5], Luca Maria Aiello[6], and Andrea Baronchelli[1,2,7,*]

[1]Department of Mathematics, City University of London, EC1V 0HB, London, UK
[2]The Alan Turing Institute, British Library, 96 Euston Road, NW12DB, London, UK
[3]Technical University of Denmark, DK-2800 Kgs. Lyngby, DK
[4]Department of Neuroscience, Catholic University of the Sacred Heart, Rome, IT
[5]IBM Research, Cambridge MA, USA
[6]IT University of Copenhagen, DK
[7]UCL Centre for Blockchain Technologies, University College London, London, UK.
[*]abaronchelli@turing.ac.uk

ABSTRACT

In this Supplementary Information, we show additional information on the data cleaning, the generation of NFTs and random networks, as well as the way we perform the sales price regression and prediction. Furthermore, we present additional analyses that support our findings.

S1 Additional information on the data and methods

S1.1 Data cleaning and categorization

NFTs that share common features are grouped in *collections*, which names are cleaned and even out. The raw names, as downloaded from the selected sources, are stripped by any digits, special characters (e.g., "-"), unusual patters (e.g., "xxxxx"), and capitalized. Cleaned names are then even out by considering a list of words. For instance, the collection Aavegotchi renames all collections starting with that string of characters in Aavegotchi. Some other collections with generic names (e.g. Stuff) are called Miscellanea.

Fields considered in our analysis are: buyer address, seller address, time of the transaction, name of the collection, ID of an NFT (here simply called "NFT"), url to the NFT's digital object, type of cryptocurrency and its amount used in the transaction. Transactions with one of the former fields empty (except for the url to the NFT's digital object) are removed from the dataset. From these remaining data, the price in USD is computed considering the exchange rate of the given cryptocurrency at the day of the transaction. Note that, in this work, we use buyer or seller addresses as proxies for real identities, as commonly done in the Ethereum blockchain[1] and with the usernames[2], while in reality an individual may have multiple addresses or usernames. NFTs sharing common features, such as, digital cards of the same online game, belong to the same collection. Also, collections are assigned to one of the following six categories: *Art*, *Collectible*, *Games*, *Metaverse*, *Utility*, or *Other*. The operative definitions of these

Category	Buyers	Sellers	NFTs	Volume ($\cdot 10^6$ USD)
Art	161,423	70,623	859,570	655.62
Collectible	62,100	67,173	1,344,449	109.84
Games	151,702	192,772	2,202,432	70.77
Metaverse	12,121	10,283	47,286	68.18
Utility	2,637	1,483	7,752	8.74
Other	34,647	22,308	242,990	21.96
Total	359,561	314,439	4,704,479	935.11

Table S1. Breakdown of NFTs categories. Overall statistics of each NFT category under consideration.

Category	Description
Art	NFTs of digital artworks, such as images, videos, or GIFs
Collectible	NFTs of interest to a collector
Games	NFTs used n competitive games
Utility	NFTs for specific purposes (e.g. secure and decentralized name service)
Metaverse	Piece of virtual worlds
Other	NFTs of small collections that are not included in the other categories

Table S2. NFTs categorization. Operative definitions of NFTs categories.

categories are inspired from the definitions given by NonFungible Corporation[3], a specialized company that track NFTs sales, and OpenSea[4], one of the largest NFT marketplace, and summarized in Table S2.

All collections with high trading volume or large number of sales were categorized by at least two authors of the present manuscript. The manual categorization was done independently by each author, then the final category selected by majority voting, asking the opinion of additional authors in case of draw between two or more categories. Note that a collection may belong to more than one category and forcing each collection into one category only is a limitation of the present work.

With the exception of the Atomic API, the downloaded datasets are not independent and, for instance, some transactions shared by NonFungible Corporation are available from OpenSea as well. When data are merged together, duplicated transactions are removed by prioritizing (in order) data from NonFungible Corporation, CryptoKitties sales, Gods-Unchained API, Decentraland API, and OpenSea API.

S1.2 Generation of the traders and NFTs networks of interaction

Example | **Trader network** | **NFT network**

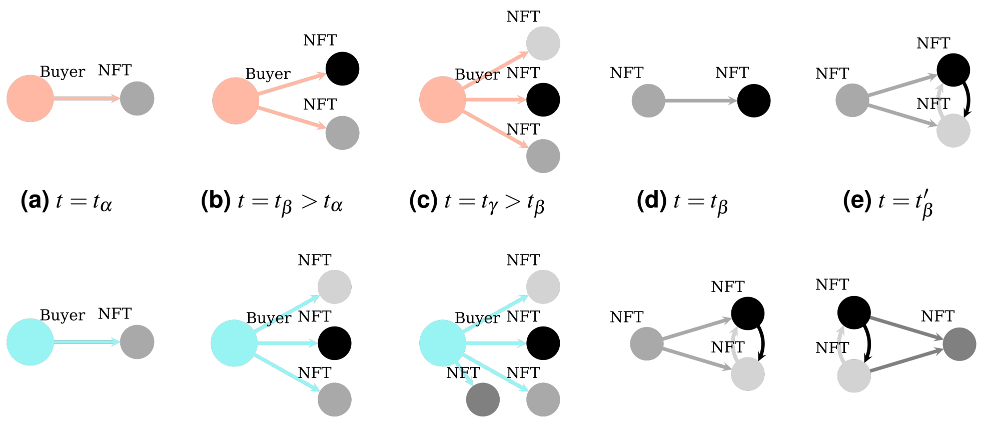

(i) — **(a)** $t = t_\alpha$ **(b)** $t = t_\beta > t_\alpha$ **(c)** $t = t_\gamma > t_\beta$ **(d)** $t = t_\beta$ **(e)** $t = t'_\beta$

(ii) — **(f)** $t = t'_\alpha$ **(g)** $t = t'_\beta > t'_\alpha$ **(h)** $t = t'_\gamma > t'_\beta$ **(i)** $t = t'_\beta$ **(j)** $t = t'_\gamma$

Table S3. Link creation mechanism of the NFT network. Directed links are generated using the trader network as reference and following three rules. The first two rules take into consideration the same buyer, while the third rule another buyer, both interacting with the same three NFTs. Visualization is done using Graph-tool:[5].

While the trader network was directly obtained from our data collection, the NFT network was created by linking NFTs that are purchased in a sequential order by the same buyer. Let's consider NFT$_i$, NFT$_j$, NFT$_k$, and NFT$_h$ as identifier of generic NFTs, and t_α, t_β, t_γ, t'_α, t'_β, and t'_γ as identifiers for time instants (with a temporal resolution of seconds). Table S3 illustrates two meaningful examples of how the NFT network is created. (i) When a buyer, who

purchased NFT$_i$ at time t_α, buy NFT$_j$ at time $t_\beta > t_\alpha$, a directed link from NFT$_i$ to NFT$_j$ is created at time t_β. If the same buyer purchases NFT$_k$ at a later time $t_\gamma > t_\beta$, a directed link from NFT$_j$ to NFT$_k$ is drawn at time t_γ. (ii) When a buyer, who purchased NFT$_i$ at time t'_α buy NFT$_j$ and NFT$_k$ at the same instant $t'_\beta > t'_\alpha$, a directed link from NFT$_i$ to NFT$_j$ and another from NFT$_i$ to NFT$_k$ are drawn. If the same buyer purchases a fourth NFT$_h$ at time t'_α. The NFT network hereby constructed includes 4 657 713 NFTs out of a total of 4 704 479. The NFTs that are left out belongs to buyers who perform only one transaction. The network analysis is done by leveraging selected functions in the networkx Python package.

S1.3 Generation of random networks

Random networks relative to the trader and NFT networks are generated in a similar fashion, and by preserving each node outgoing and incoming strength. We consider the pool of observed links with repetition, that is, a link appears a number of times equal to its weight. Two links (l_1 and l_2) are randomly extracted over this pool, where node $n^1_{l_1}$ create a directed link to node $n^2_{l_1}$ and node $n^1_{l_2}$ create a directed link to node $n^2_{l_2}$. These links are swapped if the fours nodes are different. The swap consists in creating link l'_1, where node $n^1_{l_1}$ create a directed link to node $n^2_{l_2}$, and link l'_2, where node $n^1_{l_2}$ create a directed link to node $n^2_{l_1}$. We repeat the procedure for a number of times equal to the total links in the network. We create 100 independent realization of this random network for the trader network and 100 for the NFT network.

S1.4 NFT features

We characterize NFTs with a set of 11 features, partitioned in three groups. An NFT's features were calculated only from the data that could be collected until the day before its primary sale, t_s. We used these features in two separate tasks of regression (Section S1.5), and classification (Section S1.6).

The first group of features includes network centrality scores obtained from the trader network. Specifically, we considered the degree centrality (k), and the PageRank centrality (PR) of the seller and the buyer, for a total of 4 features. The degree centrality of a node is the count of all its incoming and outgoing unique links[6], and its PageRank centrality measures the stationary probability that a random walk on the network ends up in that node[7].

The second group includes the visual features of the object associated with the NFT, namely 5 PCA components extracted from the AlexNet vector of the object ($PCA_{1...5}$). We experimented with a number of components varying from 2 to 10, and results varied only slightly—fewer components caused a feeble decrease in the quality of the regression and prediction results, while additional components did not add any predictive power.

The third and last group includes two features to account for the previous sale history in the NFT's collection. The first is the median price of primary and secondary sales made in the collection of interest during a time window prior to t_s. The latter models the prior probability of secondary sale. We acknowledge that the likelihood that a NFT gets transacted in a secondary sale might depend on the collection it belongs to. For example, NTFs corresponding to collectible items from very popular collections may be more likely to be resold than an NFT serving for a specific purpose, such as determining the ownership of a name server. We defined the probability of secondary sale, p_{resale}, as 0.5 (random probability) when the NFT is the first to be sold in its collection; else, the probability of secondary sale is calculated as:

$$p_{resale} = \frac{0.5}{n+1} + \frac{n}{n+1}\frac{s}{n},$$

where n represents the NFTs with a primary sale up to the day before the first purchase and s the number of these NFTs with at least one secondary sale. When the collection is large, the probability of secondary sales becomes $p\,(n \to +\infty) = s/n$ and corresponds to the ratio between items with secondary sales over all items with one sale.

The frequency distributions of our features have different skews and ranges. To make them comparable and suitable for regression and prediction tasks, we first transform their values to make their distributions closer to a Normal distribution. Specifically, we calculate the logarithm of the network degree and the median sale price (after adding 1, so that zero-values were preserved), and we apply a BoxCox transformation[8] to the PageRank centrality and to p_{resale}; BoxCox uses power functions to create a monotonic transformation that stabilizes variance and makes

the data closer to a normal distribution. No transformation was needed for the PCA features. Last, we scale all the variables in the range $[0,1]$ (i.e., min-max scaling).

S1.5 Sale price regression

We perform linear regressions to estimate an NFT's primary and secondary sale prices. Linear regression is an approach for modeling a linear relationship between a dependent variable (secondary sale price, in our experiments) and a set of independent variables (features describing the NFT at the time it was first sold), and it does so by associating a so-called β-coefficient with each independent variable such as the sum of all independent variables multiplied by their respective β-coefficients approximates the value of the dependent variable with minimal error. Specifically, we used an Ordinary Least Squares regression model to estimate coefficients such that the sum of the squared residuals between the estimation and the actual value is minimized.

We use the NFT features described in Section S1.4 as independent variables, and either the price of primary sale or the median secondary sale price calculated over a time window starting at t_s as dependent variables. For secondary sale price, the results changed only slightly when using different aggregations other than the median (e.g., mean, maximum). We experimented with different lengths of the time window, ranging from one week after the primary sale up to two years after. To make sure that the secondary sale price of each NFT was calculated over time windows of equal length, we exlcuded from the regression NFTs that were sold for the first time too recently—namely those NFTs whose t_s was within one time window before the most recent timestamp in our dataset. In the regression, we considered only NFTs with at least one secondary sale in the time window considered.

We evaluated the goodness of the linear fit using coefficient of determination R^2, a score in the range $[0,1]$ that measures the proportion of the variance in the dependent variable that the linear model is able to predict from the independent variables. In particular, we used its 'adjusted' version R^2_{adj}, that discounts the effect of the R^2 spuriously increasing as more independent variables are added to the model.

S1.6 Secondary sale prediction

We performed a binary classification task to predict whether an NFT will be transacted in a secondary sale after its primary sale at time t_s. We adopted a standard supervised learning approach. In supervised learning, instances in a dataset (the NFTs) are described with a number of features (those presented in Section S1.4) and marked with a target label (1 if the NFT was transacted in a secondary sale, 0 otherwise). A mathematical model learns a function that maps the features to the target label based on a number of *training* instances from the dataset. The performance of the model is later assessed on a *test* set of unseen instances. In our experiments, we emulate a prediction on future data based on past knowledge. To do so, we sort the NFTs according to their time of primary sale t_s, and we use the first 95% of NFTs for training and the latest 5% for testing. Our dataset is sufficiently large so that the test set, albeit small in relative terms, includes a large selection of tens of thousands of instances. Similar to the regression task, we consider multiple time windows of varying size to determine the target label (i.e., whether the NFT was resold or not), and we exclude from the dataset recent NFTs whose t_s is within one time window before the last timestamp in our dataset.

There are several classes of models that can be used for supervised learning[9]. We pick AdaBoost[10], an ensemble of weak learners (in our case, decision trees) whose output is combined into single score through a weighted sum. In particular, we initialized the AdaBoost classifier with 100 decision tree stumps (i.e., trees of depth 1), and trained it with a learning rate of 1. Despite its relatively simple design, AdaBoost can achieve good performance compared to more complex model and it effectively limits overfitting the learned function on the training data.

The labels of our dataset are *imbalanced*: the number of negative labels is much higher than the number of positive ones (i.e., 80% of NFTs in our dataset are more not resold). Imbalanced datasets can affect the ability of the model to learn a function that can effectively associate the correct label to both positive and negative instances. To mitigate this problem, we perform random oversampling[11] to balance the classes. Specifically, within the training set, we add multiple copies of positive samples picked at random until the size of the two classes is balanced. Compared to other oversampling techniques[12,13], random oversampling does not generate synthetic data points, which exhibiting unrealistic features. By applying oversampling, we effectively set the model to assign higher

importance to positive samples: misclassifying a positive instance causes a loss in performance that is proportional to the number of its replicas.

To evaluate the performance on the test set, we measure two quantities. The first is the *F1-score*, namely the harmonic mean of the precision (fraction of instances that are classified as positive that are indeed positive) and recall (fraction of positive instances the are correctly classified). The second is the “Area Under the ROC Curve” (AUC); it measures the ability of the model to correctly rank positive and negative samples by confidence score, independent of any fixed decision threshold. AUC is equal to 0.5 for a random classification and it is equal to 1 for a perfect ranking.

S2 Additional Analyses

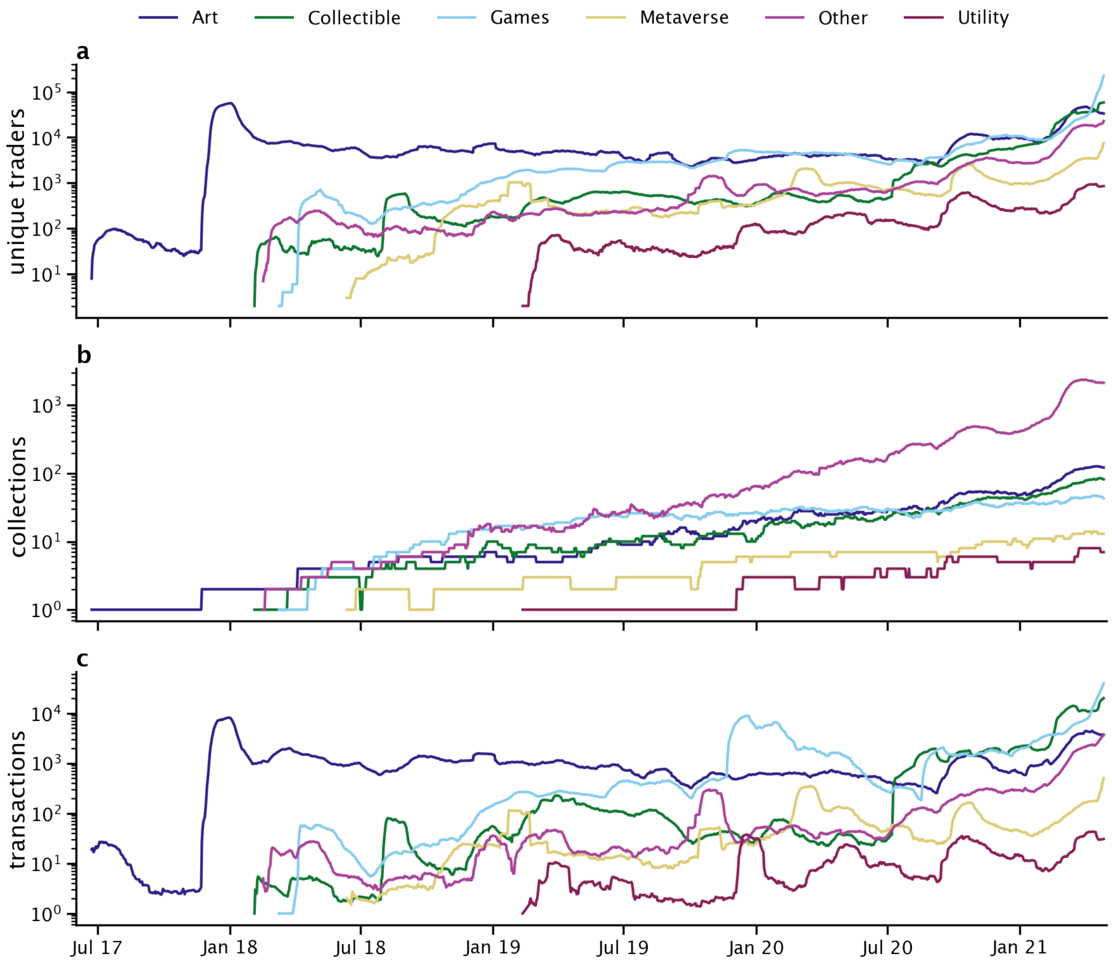

Figure S1. Evolution of the NFT market. The number of unique traders **(a)**, collections **(b)** and transactions **(c)** over time for different categories. Results are computed over a rolling window of 30 days.

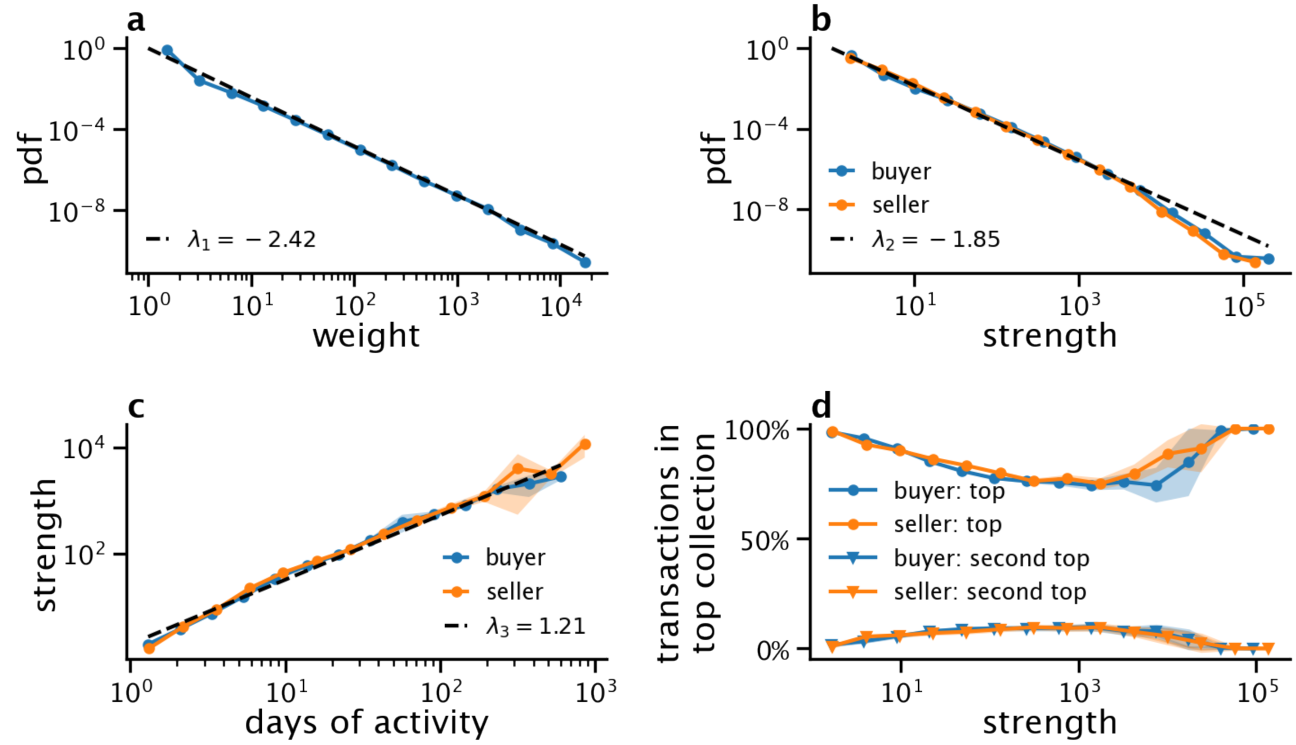

Figure S2. **Key network properties of buyers and sellers.** **(a)** Probability distribution function of the number of transactions (weight) from buyers to sellers. **(b)** Probability distribution function of the buyers and sellers' strength. **(c)** Relationship between the buyers and sellers' strength and the number of days in which they are active. **(d)** Percentage of transaction buyers and sellers make toward their top and second-top NFT collections.

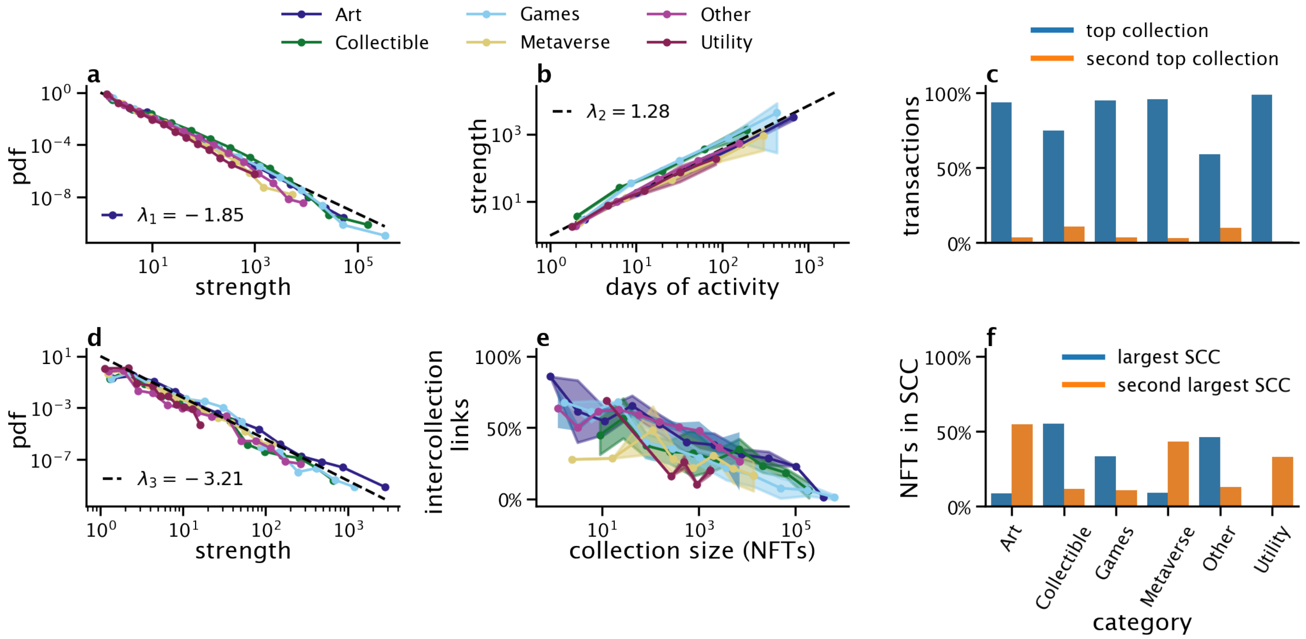

Figure S3. **Key network properties for each category. (a)** Probability distribution function of the traders' strength. **(b)** Relationship between the traders' strength and the number of days in which they are active. **(c)** Percentage of transactions all traders make toward their top and second-top NFT collections. **(d)** Probability distribution function of the NFTs' strength. **(e)** Percentage of transactions between NFTs in different collections as a function of the size of the collection. **(f)** Percentage of NFTs belonging to the first and second largest strong connected component (SCC). Solid curves in panels **(b)-(e)** represent average values, while respective bands the 95% confidence interval.

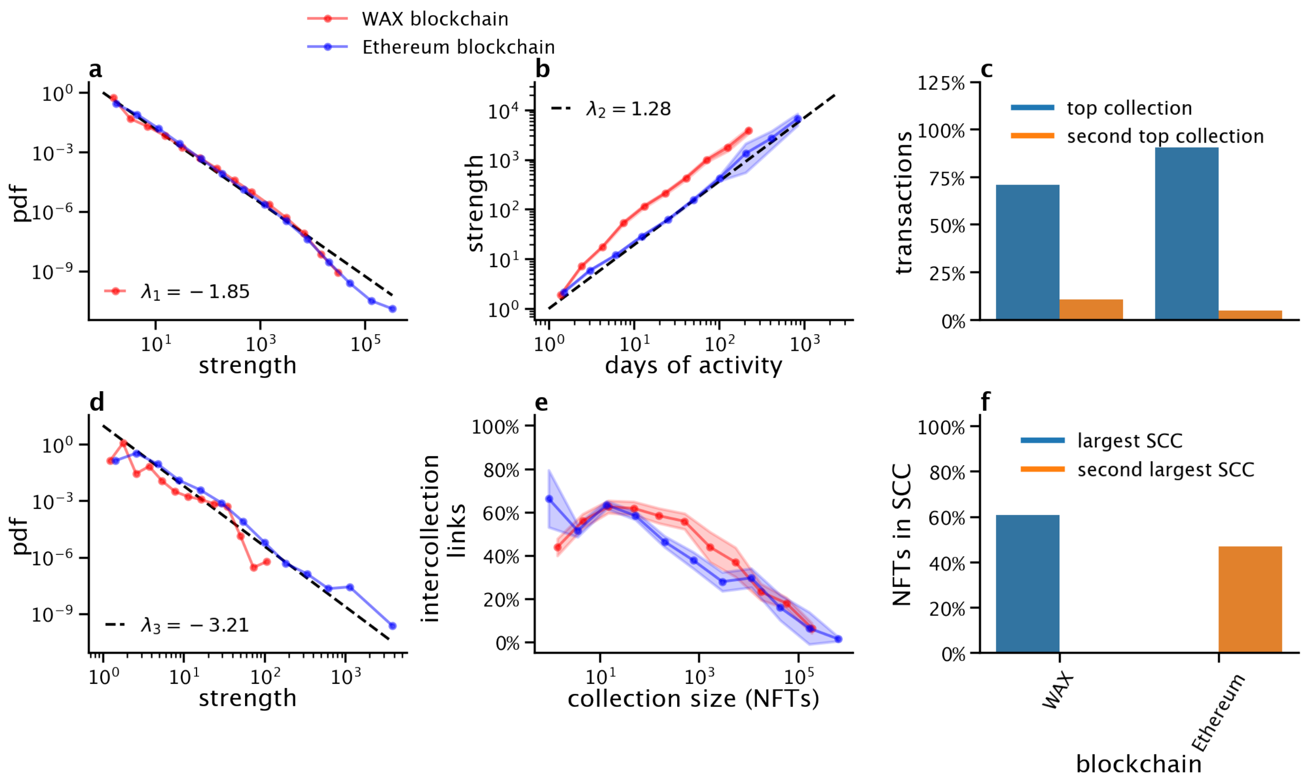

Figure S4. **Key network properties for each blockchain.** **(a)** Probability distribution function of the traders' strength. **(b)** Relationship between the traders' strength and the number of days in which they are active. **(c)** Percentage of transactions all traders make toward their top and second-top NFT collections. **(d)** Probability distribution function of the NFTs' strength. **(e)** Percentage of transactions between NFTs in different collections as a function of the size of the collection. **(f)** Percentage of NFTs belonging to the first and second largest strong connected component (SCC). Solid curves in panels **(b)**-**(e)** represent average values, while respective bands the 95% confidence interval.

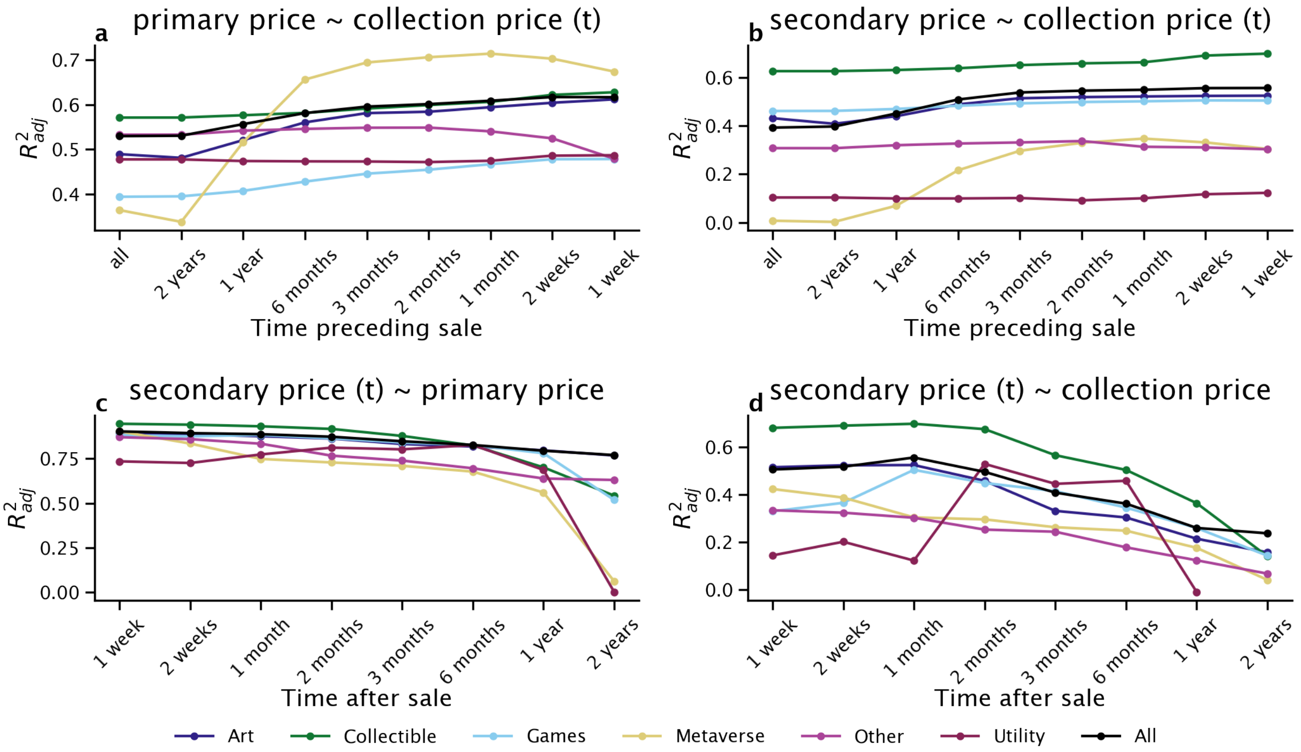

Figure S5. Primary and secondary sale price predictions. Top: R^2_{adj} of a linear regression fit to predict **(a)** the price of primary sales, and **(b)** the median price of secondary sales 1 month after their respective primary sale from the historical median price of sale in the collection calculated over varying time windows (one week to two years) preceding the primary sale. Bottom: R^2_{adj} of a linear regression fit to predict **(c)** the price of secondary sales from the price of their respective primary sales, and **(d)** the price of secondary sales from the median price of sales in the NFT's collection in the previous week; we perform different regressions to predict the median price of secondary sales over varying time windows (one week to two years) after the primary sale. All results are broken down by NFT categories.

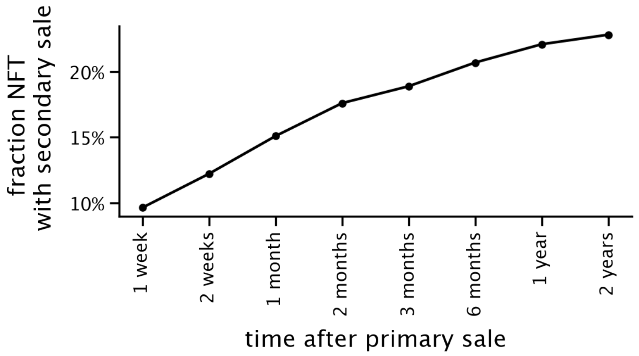

Figure S6. Vast majority of NFTs does not have a secondary sale. Fraction of NFTs that were sold in at least one secondary sale n days after their primary sale.

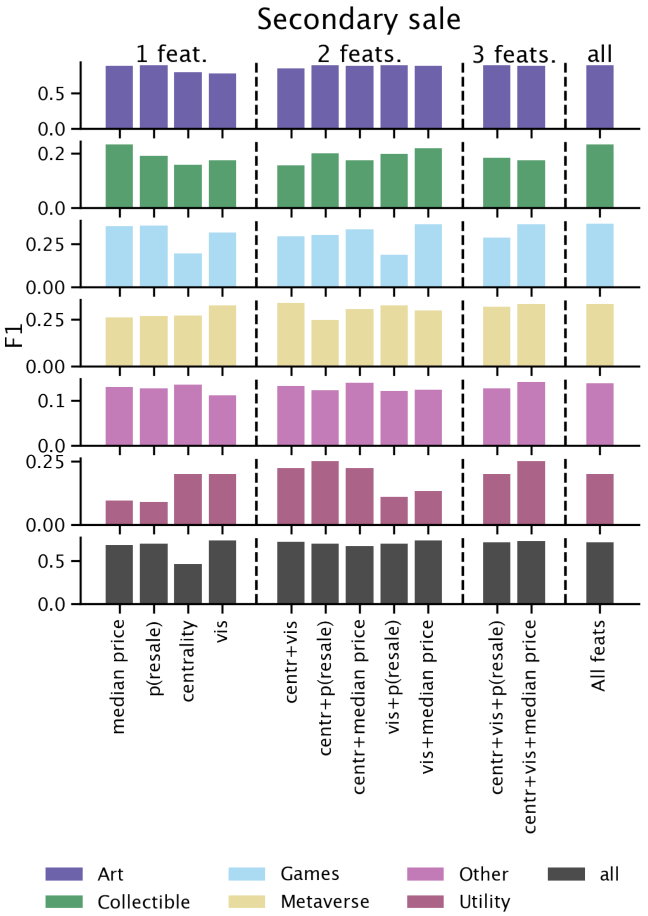

Figure S7. **Result of predicting the existence of a secondary sale.** $F1$ score of a binary classification task aimed at predicting whether a NFT will be sold in a secondary sale within 1 year after its primary sale. Results are broken down by different feature sets and NFT categories.

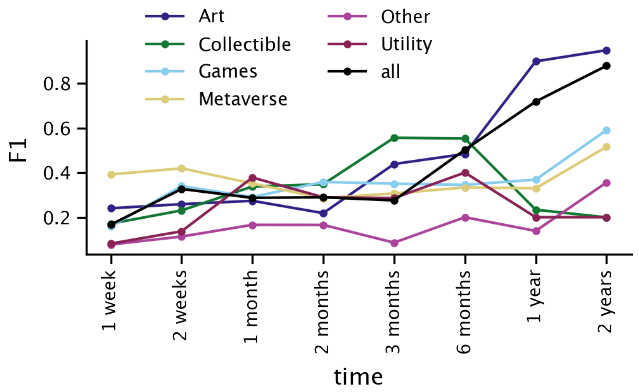

Figure S8. **Classification task with different time windows.** $F1$ score of a binary classification task aimed at predicting whether a NFT will be sold in a secondary sale within varying time windows after its primary sale. We used all available features for training and testing the models. Results are broken down by different NFT categories.

References

1. Team, L. L. All CryptoPunk owners. https://www.larvalabs.com/cryptopunks/leaderboard Accessed May 17, 2021 (2021). The New York Times.
2. Barabási, A.-L. The Art market often works in secret. Here's a look inside. https://www.nytimes.com/2021/05/07/opinion/nft-art-market.html Accessed May 17, 2021 (2021). The New York Times.
3. Team, N. The best place to analyze, track, and discover NFTs. https://nonfungible.com/ Accessed May 4, 2021 (2021). NonFungible Corporation.
4. OpenSea, T. Discover, collect, and sell extraordinary NFTs. https://opensea.io/ Accessed May 28, 2021 (2021). OpenSea.
5. Peixoto, T. P. The graph-tool python library. *Figshare* DOI: 10.6084/m9.figshare.1164194 (2014).
6. Wasserman, S., Faust, K. *et al. Social network analysis: Methods and applications* (Cambridge University Press, 1994).
7. Brin, S. & Page, L. The anatomy of a large-scale hypertextual web search engine. *Comput. Networks ISDN Syst.* **30**, 107–117 (1998).
8. Box, G. E. & Cox, D. R. An analysis of transformations. *J. Royal Stat. Soc. Ser. B (Methodological)* **26**, 211–243 (1964).
9. Hastie, T., Tibshirani, R. & Friedman, J. *The elements of statistical learning: Data mining, inference, and prediction* (Springer Science & Business Media, 2009).
10. Freund, Y., Schapire, R. & Abe, N. A short introduction to boosting. *Journal-Japanese Soc. For Artif. Intell.* **14**, 1612 (1999).
11. Ling, C. X. & Li, C. Data mining for direct marketing: Problems and solutions. In *Kdd*, vol. 98, 73–79 (1998).
12. Chawla, N. V., Bowyer, K. W., Hall, L. O. & Kegelmeyer, W. P. Smote: Synthetic minority over-sampling technique. *J. Artif. Intell. Res.* **16**, 321–357 (2002).
13. He, H., Bai, Y., Garcia, E. A. & Li, S. Adasyn: Adaptive synthetic sampling approach for imbalanced learning. In *2008 IEEE International Joint Conference on Neural Networks (IEEE World Congress on Computational Intelligence)*, 1322–1328 (IEEE, 2008).
